# Uncovering The Role of Oxygen in Ni-Fe(O_x_H_y_) Electrocatalysts using In situ Soft X-ray Absorption Spectroscopy during the Oxygen Evolution Reaction

**DOI:** 10.1038/s41598-018-37307-x

**Published:** 2019-02-06

**Authors:** Dorian Drevon, Mikaela Görlin, Petko Chernev, Lifei Xi, Holger Dau, Kathrin M. Lange

**Affiliations:** 1 Operando Characterization of Solar Fuel Materials, Helmholtz-Zentrum Berlin für Materialien und Energie GmbH, 12489 Berlin, Germany; 2 Free University of Berlin, Department of Physics, Arnimallee 14, 14195 Berlin, Germany; 3 Universität Bielefeld, Physikalische Chemie, Universitätsstr. 25, 33615 Bielefeld, Germany

## Abstract

In-situ X-ray absorption spectroscopy (XAS) at the oxygen K-edge was used to investigate the role of oxygen during the oxygen evolution reaction (OER) in an electrodeposited Ni-Fe(O_x_H_y_) electrocatalyst in alkaline pH. We show the rise of a pre-peak feature at 529 eV in the O K-edge spectra, correlated to the appearance of a shoulder at the Ni L_3_-edge and formation of oxidized Ni^3+/4+^-O. Then, for the first time, we track the spectral changes in a dynamic fashion in both the soft and hard X-ray regimes during cyclic voltammetry (in situ CV-XAS) to obtain a fine-tuned resolution of the potential-related changes. The pre-peak feature at the O K-edge likely signifies formation of an electron deficient oxygen site. The electrophilic oxygen species appears and disappears reversibly in correlation with the Ni^2+^ ↔ Ni^3+/4+^ process, and persists during OER catalysis as long the metal is oxidized. Our study provides new insight into OER electrocatalysis: Before onset of the O-O bond formation step, the catalytic oxyhydroxide has accumulated electron deficiencies by both, oxidation of transition metal ions and formation of partially oxidized oxygen sites.

## Introduction

Reducing global carbon emissions will require efficient catalysts for use in solar-to-fuel conversion processes, where electrochemical water oxidation is the key to approach zero emissions^1^. For this, understanding the fundamental processes of the oxygen evolution reaction (OER, 4OH^−^ → O_2_ + 2H_2_O + 4e^−^) from a mechanistic perspective is an important sub-goal for the design of highly efficient and functional electrocatalysts^2,3^. Ni-Fe oxyhydroxide (O_x_H_y_) electrocatalysts are currently the most active catalysts, and catalyze OER in alkaline media at low overpotentials with high O_2_ efficiency and turnover rates^4–17^. Combining Ni and Fe leads to an excessive increase in the catalytic activity, coincident with changes at the redox-active metal center, witnessing the electronic interaction between Ni and Fe sites^7,18^. The complexity of this interaction includes a modulation of the metal redox activity, which complicates the interpretation of the factors that scales with the OER activity. This have resulted in a controversy regarding the catalytically active structural and electronic state in mixed Ni-Fe catalysts^7,14,18,19^.

Friebel *et al*.^14^ presented in *operando* XAS investigations in combination with DFT + U calculations of mixed electrodeposited Ni-Fe catalysts with different compositions, which concluded that OER proceeds at lowest overpotential at Fe-sites embedded in the Ni(OOH) lattice. This study demonstrated in agreement with other studies that the active metal redox states can be described as Ni^3+/4+^-Fe^3+ ^^10,20,21^, however, according to work from us and other groups OER can also proceed on low-valent Ni^2+^ sites^15,19^. The same discussions have been held for the Fe-site where both Fe^3+ ^^14,20^ and Fe^4+^ sites^19,21^ have been observed. More recently, spectral fingerprints of Fe^6+^ sites were observed in a spectroscopic study in non-aqueous solvent by Hunter *et al*.^22^. This reveals an intriguing complexity of the catalytic system that may mask the true origin of the activity enhancement in binary Ni-Fe centers. Burke *et al*.^23^ recently presented a study with Fe-spiked KOH electrolyte that revealed two distinct catalytic sites, where the “highly-active” sites were proposed to be located at “edge” or “defect sites” since the activity was unrelated to long-term modulations of the bulk. The presence of “fast” and “slow” sites in Ni-Fe catalysts was also recently observed in a scanning electrochemical microscopy study by Ahn *et al*.^24^. The nature of the OER mechanism in alkaline electrolyte is not yet fully understood and has so far been rather simplified. The OER overpotential is assumed to be restricted by scaling relations between surface bound OER intermediates (O*, OH*, OOH*) that includes four proton-coupled electron transfer (PCET) steps resulting in high overpotential^25^. The presence of “active oxygen” species has recently been emphasized, where a non-concerted step has been proposed to occur in the mechanistic pathway by Koper and coworkers^20,26,27^. To entangle the role of the oxygen as a plausible redox-active site in Ni-Fe catalysts, further spectroscopic investigations would be required. X-ray spectroscopic investigations have so far principally been limited to the metal *K*-edges of the Ni and the Fe metal^7,14,18^, where studies involving *in situ* soft X-ray at the O *K*-edge of Ni-Fe catalysts is still lacking. This may provide useful information on the interaction and orbital mixing between the metal 3d and oxygen 2p sites^28–30^. In the only study known to us of a similar system, Yoshida *et al*. presented an *in situ* soft XAS study of an electrodeposited nickel-borate catalyst (Ni-B_i_) in near neutral pH conditions, where changes in the O *K*-edge spectra were observed as a reversible peak near 529 eV, related to formation of Ni^+3.6^ in edge-sharing NiO_6_ octahedra^31^. Using *in situ* ambient pressure XPS, Ali-Löytty *et al*.^32^ investigated the O *K*-edge of an electrodeposited Ni-Fe catalyst, however corresponding changes around the O *K* pre-edge region was absent in this study.

In this study, we present the first *in situ* measurements in both the soft and the hard X-ray regimes under OER catalytic conditions at the O *K*-edge and the metal (Ni, Fe) *L*- and *K*- edges of an electrodeposited Ni-Fe(O_x_H_y_) oxygen evolution electrocatalyst in alkaline electrolyte. We discuss the involvement of the oxygen species in OER and we further correlate changes at the O *K*-edge in a dynamic fashion with changes occurring at the metal *L*- and *K*-edges during *in situ* CV cycling.

## Experimental Section

### Sample preparation

The Ni-Fe films were cathodically electrodeposited from aqueous solution using ultrapure Milli-Q water (>18.2 MΩ cm) containing 9 mM Ni(SO_4_) × 6H_2_O (Sigma-Aldrich), 9 mM Fe(SO_4_) × 7H_2_O (Sigma-Aldrich), and 25 mM (NH_4_)_2_SO_4_. The catalyst was electrodeposited on a 150 nm thick Si_3_N_4_ membrane (Silson) coated with a 1 nm Ti adhesion layer and a 20 nm Au conductive top layer employed as working electrode. A cathodic current density of −250 mA/cm^2^ was applied for 5 s on a limited area of 2 × 2 mm, which was considered as the geometric surface area of the working electrode. The metal loading was determined by total reflection X-ray fluorescence spectroscopy (TXRF), using a benchtop S2 Picofox spectrometer (Bruker) with a Mo K_α_ source at 40 kV and a Si-drift detector. The metal loading on the electrodes was determined to 510 nmol/cm^2^ of Ni and 275 nmol/cm^2^ of Fe (Ni:Fe = 65:35). The film thickness was estimated to 400 nm by assuming 0.125 µC/cm^2^ per 1 nm^33^.

### Scanning Electron Microscopy (SEM)

The morphology of the catalyst film deposited on the Si_3_N_4_/Ti/Au was characterized using a LEO Gemini 1530 field emission scanning electron microscope (FESEM) at the Helmholtz Zentrum Berlin (HZB), of as-deposited catalysts and after the soft X-ray *in situ* OER characterization.

### *In situ* X-ray absorption spectroscopy and electrochemical measurements

**Figure 1:**
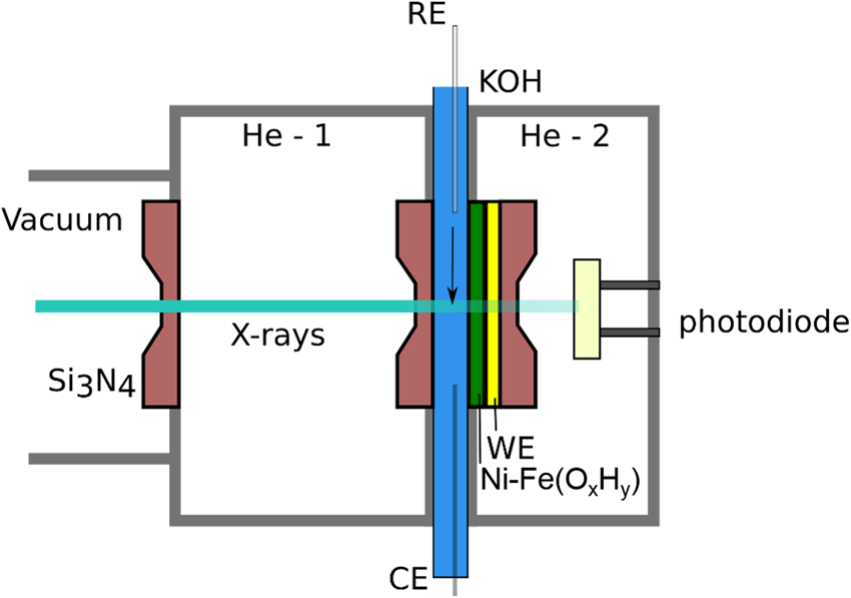
A schematic illustration of the soft X-ray transmission cell used in this study. The catalyst is deposited on the Au-coated silicon nitride membrane window closest to the photodiode. The electrolyte is confined between the two Si_3_N_4_ membranes. The GaAs photodiode measures the X-ray transmitted through the electrolyte and membranes.

*In situ* electrochemical X-ray absorption measurements of the O *K*-edge and Ni, Fe *L*-edges were performed with a PEEK transmission cell in transmission mode^34^, at the LiXEdrom 2.0 end-station at the U56-2 PGM2 beamline at BESSY II (HZB). The layout of the liquid cell used for the *in situ* measurements is illustrated in Fig. 1. Similar to previous work^35^, the thin layer of electrolyte is confined between two assembled Si_3_N_4_ membranes (100 nm). One of them is coated with 1 nm Ti, 20 nm Au and the Ni-Fe catalyst, which serves as working electrode. A Pt-wire was used as counter electrode, and a leak-free Ag/AgCl (Ø = 1 mm) applied as reference electrode. The electrochemical measurements were controlled using a Bio-Logic SP-200 potentiostat. All electrode potentials are reported on the reversible hydrogen electrode scale (E_RHE_ = 0.21 V_Ag/AgCl_ + 0.059 V × pH). The two He-chambers of the XAS cell (He-1 and He-2) were constantly purged during the measurements in order to maximize the X-ray transmission signal by removing air and water vapor. Applying He-pressure also allows to bend the two membranes so that the thickness of the electrolyte layer is reduced and thus the detrimental absorption of the soft X-ray at the O *K*-edge is decreased^36^. All measurements were carried out in 0.1 M KOH electrolyte pH 13. A syringe pump was used to keep a constant electrolyte flow of 4 µl/s. The resistance in the sXAS cell during the *in situ* measurements was estimated to ca 200 Ω. This pressure was varying with the He-pressure, whereas this was kept constant. The measurements are shown without iR-compensation.

The slit width was set to 50 µm to obtain a spectral resolution of 200 meV at an energy of 535 eV. The cell was located out of focus by 50 cm as described in our previous reports^35^. The He pressure inside the cell was kept constant at 6 kPa during the *in situ* measurements. Photons were collected using a GaAs photodiode (Hamamatsu G1127). The current was measured using a 6514 Keithley electrometer with 2 nA range sensitivity. Every spectrum was averaged on three consecutives scans and were normalized to the current produced by the beam hitting the refocusing mirror before the sample. At the O *K*-edge the spectra were calibrated using the pre-edge of water at 535 eV^37–40^ and normalized to the the NEXAFS region above 570 eV. In order to reveal the contribution of the different electronic states present in the sample, we decomposed the O *K*-edge spectrum in its spectral components using multi-peak fitting in Matlab, similar to the procedure followed by Guo *et al*.^41^. For more details, see Supporting Information. The Ni and Fe *L*-edges were calibrated using the L_3_ peaks of NiO at 852 eV^42^ and that of Fe_2_O_3_ at 708.5 eV^43^. A linear background was subtracted and the intensities were normalized to the maximum intensity of the L_3_ edge. To avoid radition damage, we significantly reduced the exposition of the catalyst to X-ray by selecting an experimental geometry where the incoming photons are attenuated by the electrolyte solution before hitting the catalyst sample. In order to ensure the reproducebility of the measurements, 3 repetitions were carried out for each configuration.

*In situ* XAS in the hard X-ray regime at the Ni and Fe *K*-edges were carried out at the KMC-3 beamline (BESSY II, HZB). A silicon (111) double-crystal monochromator was used for selecting a fixed X-ray excitation energy. The electrochemical cell was made of PTFE, and the Ni-Fe catalyst electrodeposited on Au-coated glassy carbon working electrodes (area 0.2 cm^2^). A Pt-mesh was used as counter electrode, and a leak free Ag/AgCl filled with 3 M KCl as reference electrode. The resistance in the cell was estimated to ca 30 Ω. The sample was placed 45 °C to the incoming X-ray beam using back-side illumination, and the fluoresence monitored using a scintillation detector. The amplified signal was recorded with a Bio-Logic SP-200 potentiostat. For more details, see reference^44^.

### Cyclic Voltammetry during X-ray absorption spectroscopy

Cyclic voltammetry-X-ray absorption measurements (CV-XAS) was performed at the U56-2 PGM2 and KMC-3 beamlines for the sXAS (O *K* and Ni, Fe *L*) and for the hard XAS (Ni, Fe *K*), respectively. The energy of the beam was set to a constant value of 854.1 eV for the Ni *L*, 529 eV for O *K*, 842 eV for Ni *K*, and 726 eV, and for Fe *K-*edges. The scan-rate was set to 5 mV/s or 10 mV/s while the Keithley signal was recorded with an analog-to-digital (AD) converter using the provided EC-lab software from Bio-Logic. The photons were collected with a photodiode and the resulting current was read from the output of the Keithley. The shown CV curves is an average of at least 16 individual CV cycles between ~1–1.7 V vs RHE.

## Results and Discussion

### Electrochemical Properties and Surface Morphologies

An electrodeposited Ni-Fe oxyhydroxide (O_x_H_y_) catalyst was investigated for the oxygen evolution reaction using *in situ* X-ray absorption spectroscopy in the soft and hard X-ray regimes. SEM characterization showed that the Si_3_N_4_/Ti/Au electrode substrate was decorated with platelet-like sheets, with a structure similar to the ones reported for other electrodeposited Ni-Fe oxyhydroxide catalysts (see Supporting Information Figure S1a)^19,45,46^. The Ni-Fe samples examined after the XAS investigations (after OER characterization) showed similar structure as the freshly as-deposited catalyst (see Fig. 1b). Total reflection X-ray fluorescence spectroscopy (TXRF) confirmed that the catalyst had a Fe-content of 35 atomic %. Measurements of an empty electrode substrate in comparison to the substrate with the electrodeposited Ni_65_Fe_35_ catalyst confirmed a negligible OER activity of the bare glassy carbon substrate, confirming that the OER activity originates from the Ni_65_Fe_35_ electrocatalyst (see Figure S2).

### *In-situ* X-ray absorption spectroscopy at the nickel and iron L- and K-edges

The Ni_65_Fe_35_ catalyst was investigated using soft X-ray (sXAS) at the Ni and Fe *L*-edges at several electrode potentials. The applied potential was increased by steps of 0.05 V into the OER region. For 3d transition metals, sXAS at the metal *L*-edge directly measures the unoccupied TM-3d states through dipole-allowed 2p-to-3d transitions.

**Figure 2:**
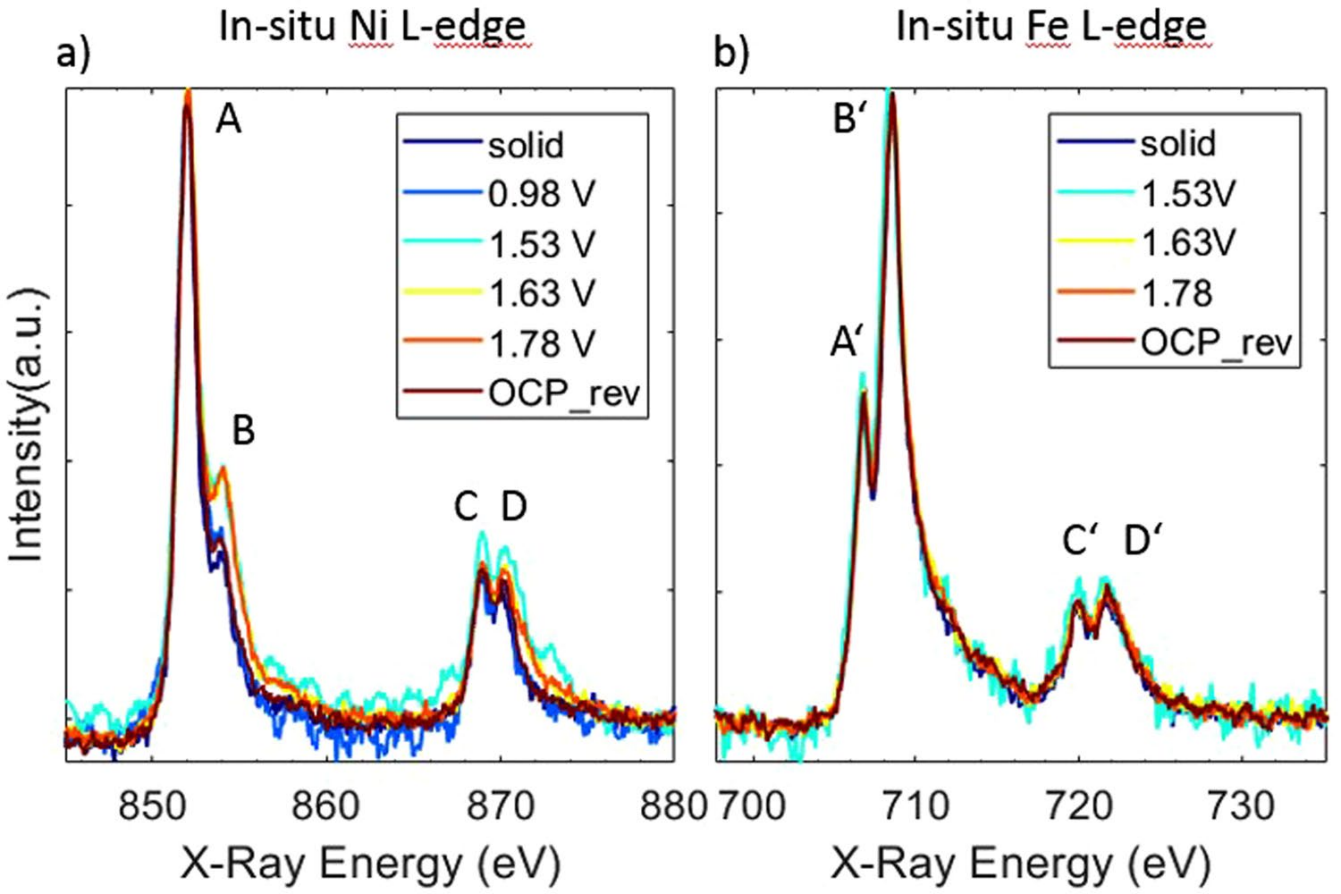
*In Situ* XAS of electrodeposited Ni-Fe catalyst at (**a**) the Ni L-edge and (**b**) Fe L-edge. Potential values are in V vs RHE, where “rev” means “reverse” direction when stepping from higher to lower electrode potentials, and “OCP” stands for open circuit potential. The measurements were carried out in 0.1 M KOH, and the potentials are reported on the RHE scale.

The *in situ* sXAS spectra at the Ni *L*-edges are shown in Fig. 2a. The electrochemical activity during the measurement is shown in the Supporting Information Figure S3. The overall spectral line shape consists of features in two regions, *L*_3_-edge around 852 eV (features A and B) and *L*_2_–edge around 870 eV (features C and D), resulting from the core- hole spin-orbital-coupling split. The fine splitting in both *L*_3_ and *L*_2_ is due to the crystal field effect from local ligand environment^47,48^. Those features result from dipole transitions from the 2p core level of the metal to empty states taking atomic multiplet effects into account which are sensitive to the electronic and oxidation state of the metal, and to the local geometry^47^. Upon increasing the potential from 0.98 V to 1.53 V vs. RHE, the amplitude of feature B is intensified. When the potential is reversed the amplitude of feature B drops down to its initial value. The intensity ratio of the double-peak features in the Ni *L*_3_ region (at 852 and 854.1 eV) fingerprints the oxidation state of the Ni atoms in the catalyst^49,50^. Our measurements do not support formation of Ni^4+^ due to the absence of a shift in the position of the enhanced feature B, i.e. the 2^nd^ shoulder of the *L*_3_ doublet feature. However, the rise of feature B during OER conditions reveals the presence of Ni species in oxidation state +3, showing that the Ni_65_Fe_25_ catalyst oxidized from Ni^2+^ → Ni^3+ ^^51^. This observation is in accordance with previous reports of oxidation states obtained from Ni *K*-edge measurements of mixed Ni-Fe catalysts during OER catalytic potential^7,14,50^.

In Fig. 2b, the Fe *L*-edges are shown, with the *L*_3_-edge peak around 707 eV (features A’ and B’) and the *L*_2_-edge peak around 720 eV (features C’ and D’). In contrast to the Ni *L*-edge, there is no observed potential dependence of the spectral features on the Fe *L*-edge, hence our measurements are compatible with Fe^+3^ both during non-catalytic and OER catalytic potential. This observation is in agreement with previous studies at the metal *K*-edge of Ni-Fe catalysts^7,14^. Oxidation states of Fe^4+^ or higher have on the other hand been reported during OER^18,20,21^. We cannot rule out that a small fraction of surface related Fe atoms may enter a higher oxidation state in our catalysts, however if the Fe atoms that changes oxidation state are a minority in number (for example appear at edge or defect sites) they may not be possible to resolve in these measurements. It is also possible that the Fe species formed during OER are too reactive to be observed under the current reaction conditions, in line with previous discussions regarding the reactivity and equilibrium states of intermediates occurring in the catalytic cycle^7,22^.

To support our observations at the metal *L*-edges, we performed *in situ* XAS measurements in the hard X-ray regime at the Ni and Fe *K*-edges, where the spectral fingerprints are well known and oxidation states can be extracted from the edge positions. These measurements showed that the Ni oxidation process begin at 1.48 V vs. RHE, seen as an edge shift to higher energies (see Figure S4a). The Fe *K*-edge on the other hand did not undergo a pronounced edge shift, however a small visible change in the white line intensity occurred at electrode potentials above the threshold of Ni oxidation, in accordance with other work on Ni-Fe catalysts during *in situ* conditions (see Figure S4b)^7,9,14,21^. It has been discussed whether this corresponds to an oxidation state change at the Fe site or a change in the geometry as a response to the oxidation state increase at the Ni site during OER^9,14,21^. In accordance with these studies, the changes we observe in the white line intensity may indicate that a small fraction of Fe atoms enters a higher oxidation state in our mixed Ni-Fe catalyst.

The Ni *K*-edge showed an edge shift of +1.2 eV at the highest measured potential, which corresponds to an oxidation state increase of 0.7 units, determined using calibration curves with known reference compounds^7^. This is in agreement with the transition from Ni^2+^ to Ni^3+^, hence confirming the observations at the Ni *L*-edge. From the Ni *K* pre-edge, contributions from a metallic phase was evident in our Ni_65_Fe_35_ catalyst, which was also reported in a recent study of a similar electrodeposited Ni-Fe catalyst^9^. Using linear combination of reference compounds, we could correct for this metallic contribution (see Figure S5a,b). The oxidation state increase of the pure oxide phase (after subtraction of the metallic phase) was estimated to 1.1 units, so slightly higher oxidation state than without corrections for the metallic contribution. This is still compatible with Ni^3+^, however we cannot exclude that a fraction of the Ni atoms enters an oxidation state higher than +3. Therefore, we conclude that the Ni atoms in our Ni_65_Fe_35_ catalyst do not reach “full” oxidation with Ni in oxidation state between +3 and +4^7,14^. We also noticed that on the reverse scan a portion of the Ni atoms needed a longer time to relax back to the initial reduced Ni^2+^ ground state, also observed in previous studies^20,52^.

### *In situ* soft X-ray absorption spectroscopy at the O *K*-edge

**Figure 3:**
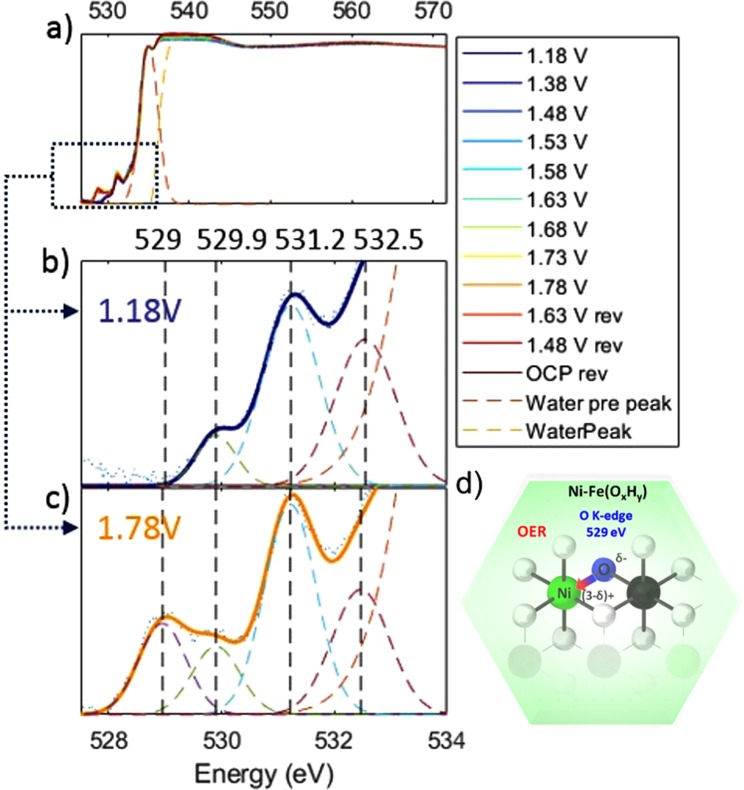
*In situ* XAS O *k*-edge spectra of electrodeposited Ni-Fe catalyst measured at various applied potentials. Potential values are given vs RHE., where “rev” means the reverse direction from higher to lower potentials. (**b**) O *K*-edge prefeature region at 1.18 V and (**c**) at 1.78 V vs RHE. Blue dots represent the experimental points, colored dashed lines the fit components of the multi-peak fitting and solid color lines the fit data model. The peak at 529 eV is the O1s-O(2p)/Ni(t2g) transition and appears at OER potential due to the increase in oxidation state of Ni. The measurements were carried out in 0.1 M KOH. (**d**) Schematic illustration showing the correlation between the changes at the O *K*-edge at 529 eV and the Ni *L*-edge at 854.1 eV; a partial donation of the higher electron density on the oxygen site (O^δ−^) to the Ni metal site (O → Ni charge transfer).

X-ray absorption spectroscopy at the O *K*-edge was used to probe the local bonding and symmetry properties of the X-ray excited oxygen atom. The O1s spectra of the electrodeposited Ni-Fe catalyst under applied potential are shown in Fig. 3a. The O *K*-edge spectra can be divided into three distinct regions. The high-energy region above 540 eV corresponding to the extended X-ray absorption fine structure. The spectra presented here were normalized to the intensity in this region.

The low energy region between 525 eV and 534 eV is the region of main interest in this work and a detailed view is presented in Fig. 3b,c. This low energy region typically originates from electronic transitions from the O1s shell to transition metal 3d orbitals hybridized with oxygen 2p orbitals^28,48,53–55^. Four peaks could be distinguished at 529 eV, 529.9 eV, 531.2 eV and 532.5 eV. We assign these to the transitions from O(1 s) to O(2p) orbitals mixed with Ni(3d)t_2g_, Fe(3d)t_2g_, O($\pi$*) of O_2_ gas, and Fe(3d)e_g_, respectively^28,56,57^. The peak positions were determined with Gaussian multi-peak fitting (see Figure S6a–f, Tables S1–S2 for fit parameters, and Table S3 for literature peak assignment). A reference spectrum of the O *K*-edge for O_2_ gas is shown in Figure S7. Spectral intensities between 534 eV and 540 eV result from near edge transitions of the water molecule, specifically at 535 eV, and at the main- and post-edge at 537.5 eV and 542 eV, respectively. Note, that main- and post-edge features are saturated. The observed intensity variations of main- and post-edge do not correlate with the applied potential but increase with measurement time (see Figure S6e,f). These changes may also be influenced by slow variations in the He pressure of the sXAS transmission cell, which can cause changes in the electrolyte layer thickness between the two assembled Si_3_N_4_ membranes. Similar changes of the pre-peak to main-peak ratio were observed for a transmission cell by Schreck *et al*.^40^, who correlated this to inhomogeneity of the liquid layer.

At increased electrode potential we observe a rise of a new pre-peak feature at 529 eV, which appears at 1.48 V and disappears reversibly at reductive potentials (see Figure 3a and S6a). We note that this peak is coincident with the oxidation of Ni^2+^ → Ni^3+^ as seen in both the Ni *L* and *K* edges. In accordance with previous studies, we correlate the 529 eV feature to the hybridization between O(2p) and Ni(3d)t_2g_ orbitals, t_2g_ being the 3d orbital with lowest energy in the frame of molecular orbital theory for an octahedral complex^28,41,48,54,56^. The other new feature located right above this peak, more specifically at 529.9 eV, is assigned to interactions between the O(2p) and hybridized Fe(3d) orbitals. This peak showed a much smaller change with the applied electrode potential in comparison to the 529 eV peak. This implies that Fe undergoes a smaller modulation, which supports the observations at the Fe *K*- edge where only a small change was observed in the white line intensity. This shows that a small modification at the Fe site is likely, however much smaller in comparison to the changes at the Ni site.

A similar pre-peak at 529 eV was reported in a soft XAS study of a Ni-B_i_ catalyst in borate buffer (near neutral pH) by Yoshida *et al*.^31^, and was assigned to bridging oxygen atoms coordinated to oxidized Ni^+3/+4^. This was explained by the oxide oxygens (O) connected to oxidized Ni(OOH) which gives rise to a O K-edge peak at lower energies in comparison to hydroxide oxygens (-OH)^31^. This pre-peak feature is indeed present in the powder reference compound LiNiO_2_, where Ni is in oxidation state +3 (see Figure S8). In work from other groups on metal oxides, the rise of a pre-peak at such low energies has been given alternative explanations. In work by Suntivich *et al*.^28^ a pre-peak in the O *K*-edge spectra was observed in perovskite OER catalysts. This was explained as an increase in hybridization between 3(d) and O(2p) states, which could cause injection/extraction of electrons from oxygen to the metal site (Ni^3+^-O^2−^ → Ni^(3-δ)+^ − O^(2-δ)−^), which was correlated to a more efficient OER activity, see Fig. 3d^28^. In work by Cho *et al*.^58^ Ni vacancies in NiO increased the formal charge on nearby atoms, which was proposed to favor charge transfer from O → Ni to stabilize a Ni^2+^-O^−^ complex^56,58^. It is therefore possible that a charge redistribution occurs in concomitance with the oxidation of Ni^2+^ to Ni^3+^. In previous *in situ* XAS studies by us we have shown that Fe-dopants in mixed Ni-Fe catalysts lowers the average oxidation state of Ni atoms during OER^15^. This was correlated to a significant decrease in structural order based on the FT-EXAFS amplitudes, which could indicate a higher number of defects. If such vacancies facilitate O → Ni charge-transfer, it may explain the observed stabilization of low-valent Ni^2+^ sites in mixed Ni-Fe catalysts.

We also note the similarity between our pre-peak and a peak reported by Pfeifer *et al*.^59–61^ in a series of *in situ* O *K*-edge and combined DFT studies of IrO_2_. They assigned a pre-peak at 529 eV to formation of oxygen in formal oxidation state −1. The nature of these O^1−^ species was experimentally confirmed to be highly electrophilic and hence likely to facilitate nucleophilic attack^61^. According to their DFT theoretical O *K*-edge spectra of IrO_2_, neither stable peroxide (O-O) nor superoxo (O-O^−^) species are likely to be represent the pre-peak at such low excitation energies as 529 eV^60^. The nature of the oxygens species related to the pre-peak was therefore thought to be singly adsorbed oxygen (O-CUS) at undercoordinated sites (µ_1_-O or µ_2_-O), which is only feasible at edge or defect sites in Ni-Fe oxyhydroxides. In a recent experimental study by Burke Stevens *et al*.^1^ of Ni-Fe catalysts prepared by spiking the electrolyte with Fe^3+^ ions, observations support that edge or defect sites are more reactive towards OER. Koper and coworkers discussed formation of “active oxygen” of superoxo character (Ni-OO^−^) during OER in NiOOH catalysts seen as a band between 950–1150 cm^−1^ in surface enhanced Raman spectroscopy, which was rising in correlation to the nickel oxidation to Ni^3+^-O(H)^26,27^. Based on this, a non-concerted proton-coupled electron transfer (PCET) step was included in the OER mechanism. In previous studies by us we also noticed a deviation from ideal Nernstian behavior of the nickel oxido redox couple in Ni-Fe catalysts, which we explained as a two proton-one electron transfer step^22^. This is in contradiction to the DFT study presented earlier by Friebel *et al*.^14^, where Fe was found to have the lowest overpotential and thus proposed as the active site, however, radicals were not discussed in the context.

Whether our observed oxygen species are formed at Ni or Fe sites is not possible to discriminate, however the largest change in metal oxidation state is observed at Ni sites. Formation of oxygen of radical character on the other hand would not force a change in the metal valency, whereby we cannot conclusively verify the site of formation. In addition, if species are formed at undercoordinated sites such as edge and defect sites it would be a minority of the total number of sites in our Ni_65_Fe_35_ catalyst.

### *In situ* cyclic-voltammetry - X-ray absorption at O *K*-edge Ni *L*-edge

**Figure 4:**
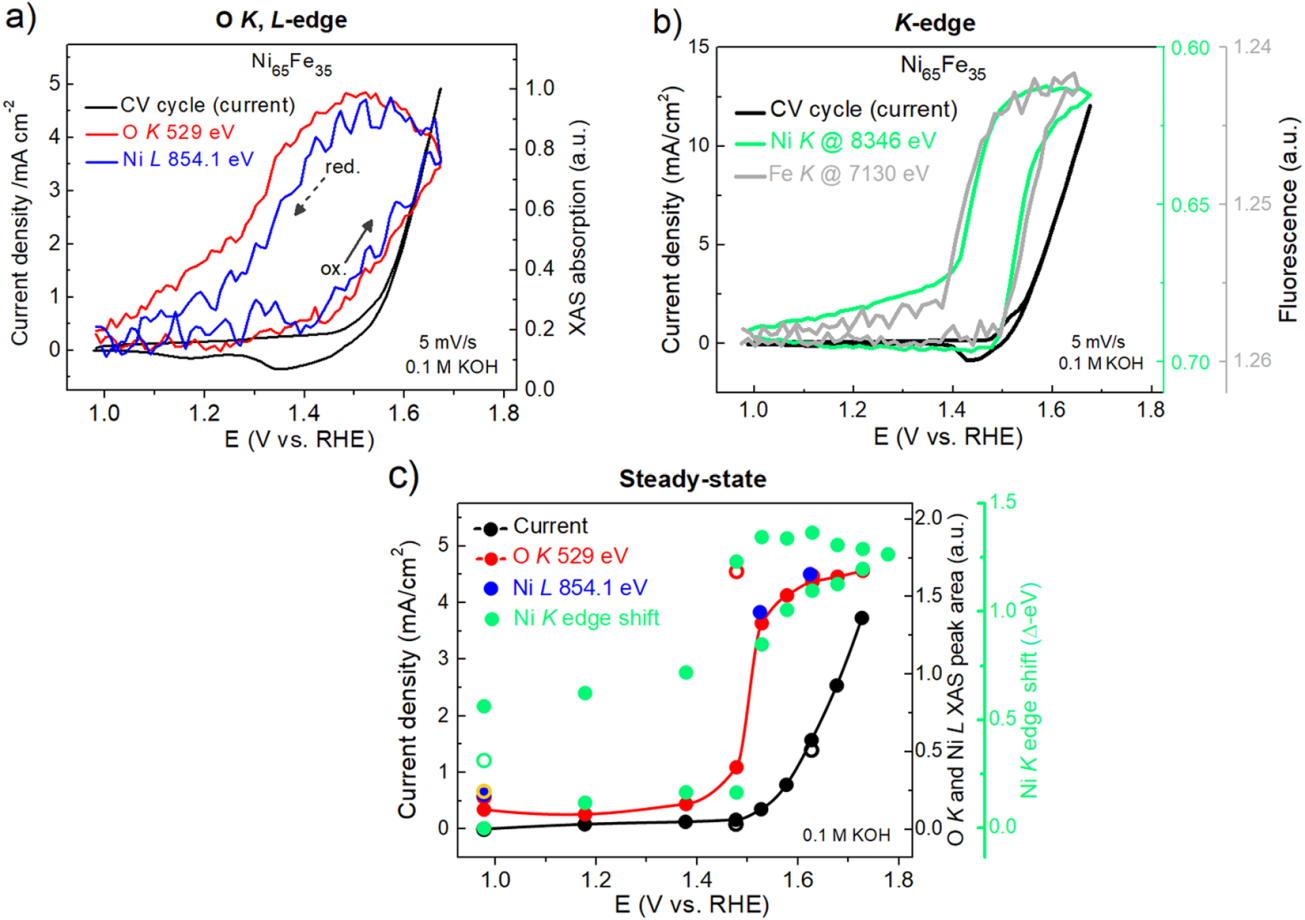
*In situ* XAS measurements of the Ni_65_Fe_35_(O_x_H_y_) catalyst in 0.1 M KOH (**a**) Soft XAS absorption intensities at the Ni L- and O *K*-edge at fixed beam energy while CV cycling between 0.98–1.68 V vs. RHE at a scan-rate of 5 mV/s. The X-ray absorption for the O *K*-edges was measured at 529 eV (red curve), and for the Ni *L*-edge at 854.1 eV (blue curve). The arrows indicate the scan direction. (**b**) The Ni *K*-edge shift at a fixed beam energy (8346 eV for Ni K and 7140 eV for Fe K, right axis) while CV cycling at 5 mV/s (left axis). The edge shift is reported as delta-eV where the baseline level was the edge position at 1.0 V vs. RHE. (**c**) Steady-state measurements showing the extracted peak areas under the corresponding O *K* peak (red) and Ni *L* peaks (blue, orange), and the edge shift at the *K*-edge (delta eV, green). The current density is shown on the left axis. The closed red, blue and black dots represent the anodic scan, the respective open dots the cathodic scan. For the Ni *K*-edge the cathodic scan runs above the anodic one and both are shown in green dots, and the open green dot is after an additional waiting time of 15 min at the end of the measurement. The Ni *L* peak was scaled to the O *K* peak for simplicity.

In order to fine-tune the changes observed in the XAS spectra with the OER activity in a dynamic fashion in our electrodeposited Ni_65_Fe_35_ catalyst, the XAS transmission signal was monitored during CV cycling at the O *K*, Ni *L*, and Ni *K* edges at a fixed beam energy. The Keithley signal was recorded via an analog-to-digital converter read directly by the Bio-Logic potentiostat software (see Fig. 4a). In the soft XAS regime, the energy was fixed either at the O *K* pre-peak energy at 529 eV or at 854.1 eV for the *L*-edge feature, since both these peaks were seen to change reversibly as a response to applied electrode potential. These were assigned to the hybridization of O(2p)-Ni(3d)e_g_ at the O *K*-edge and the Ni *L*_3_ shoulder reflecting formation of Ni^3+^, respectively. At the Ni *K*-edge, the energy was fixed at the rising main edge (8346 eV) and at the Fe *K*-edge slightly above the main edge at the white-line intensity (7130 eV) where the largest change were visible.

During the CV cycling, the absorption both at the O *K* (529 eV) and the Ni *L* (851.1 eV) peaks in the soft XAS regime were strongly correlated and followed nearly identical behavior (see Fig. 4a). Both peaks started to rise at the same potential (~1.40 V vs. RHE), which is at pre-catalytic potentials. Both traces steadily grow on the anodic scan and into the OER region, and continue to grow as the potential sweep is reversed until the potential reached the onset of the metal reduction wave. The O *K* and Ni *L*_3_ peaks then decline in correlation with the metal reduction towards the end of the CV scan. When the potential is reversed to the initial value of 0.98 V, the two signals are almost decayed back to the background levels. Evident from these measurements is that the pre-edge feature at the O *K*-edge is closely related to the changes at the Ni *L*_3_-edge peak. Hence, the Ni^2+^ → Ni^3+^ oxidation and the appearance of the O *K* pre-edge peak at 529 eV appear as two entangled processes and occur at electrode potentials prior to the onset of OER and hence prior to O-O bond coupling. This was further confirmed in the corresponding CV-XAS measurements at the Ni and Fe *K*-edges, where the oxidation state change of Ni and the modulation in white-line intensity of Fe were followed in a similar fashion (see Figs 4b and S9). These measurements confirmed that the oxidation state changes at Ni *K* edge and the changes in white line intensity at the Fe *K* edge are as well two closely related processes. Hence, the O *K* pre-edge peak at 529 eV, the oxidation of Ni^2+^ → Ni^3+/4+^, and the changes at the Fe *K*-edge are all correlated, and occurs at pre-catalytic potentials, which was also confirmed in steady-state measurements (see Fig. 4c). The correlation between the oxidation at the Ni *K* and Fe *K* edge was recently demonstrated in a study by González-Flores *et al*.^9^, where it was clear that these two processes set off prior to the onset of OER. We also take notice of the “hysteresis” with respect to the oxidized nickel seen by Yoshida *et al*.^31^, which reflects the peak separation between the oxidation/reduction peak visible in the CVs, where the reduction is more difficult than the oxidation. In our steady-state measurements it was evident that the reduction of Ni centers was slow, however if an additional waiting time of 15 min was added at the end of the measurements at 0.98 V significantly more Ni centers had relaxed (see Fig. 4c). Differences between the hard and soft X-ray regimes are therefore certain influenced by the time held at each potential.

In the previous soft XAS study by Yoshida *et al*.^31^ a Ni-B_i_ catalyst in 0.1 M potassium borate solution, the O *K*- pre-peak at 528.7 eV appeared at 0.7 V vs. Ag/AgCl (estimated by us to ~1.45 V vs. RHE assuming pH 9.2), coincident with the onset of the catalytic current. The corresponding Ni *L*- spectrum was not recorded at this potential, however, detection of Ni^4+^ was confirmed at 1.0 V vs. Ag/AgCl (~1.75 V vs. RHE) from measurements at the Ni *K*-edge^31^. Therefore, it is not possible to draw conclusions regarding the correlation between the metal oxidation and the onset of the O *K* edge pre-peak in their measurements. Interestingly, in the studies of IrO_2_ by Pfeifer *et al*.^59–61^, the theoretical simulations of the spectra concluded that the pre-peak at 529 eV assigned to O^1−^ existed only at coordinatively unsaturated sites such as µ_1_-O and µ_2_-O sites, however protonated forms of these (µ_1_-OH and µ_2_-OH) or in-plane µ_3_-O in rutile type IrO_2_ were not compatible with a pre-peak at such low energies^60^. In our electrodeposited Ni_65_Fe_35_(O_x_H_y_) catalyst, the “bulk” of the oxyhydroxide layers is mainly composed of µ_3_-O(H) sites, whereas “edge” or “defect” sites would contain such µ_1_-O and µ_2_-O in the oxidized form. With regard to recent studies showing that edge or defect sites may be more reactive towards OER^23,24^, we speculate that the 529 eV peak reflects a more reactive site located at such undercoordinated sites. This would also require that the oxidation of Ni^2+^ → Ni^3+/4+^ happen prior to the onset of OER, which we observe. This would be in line with the picture presented by Yoshida *et al*.^31^ from the O *K*-edge measurements, where it was proposed that only surface species in NiOOH participate in OER.

**Figure 5:**
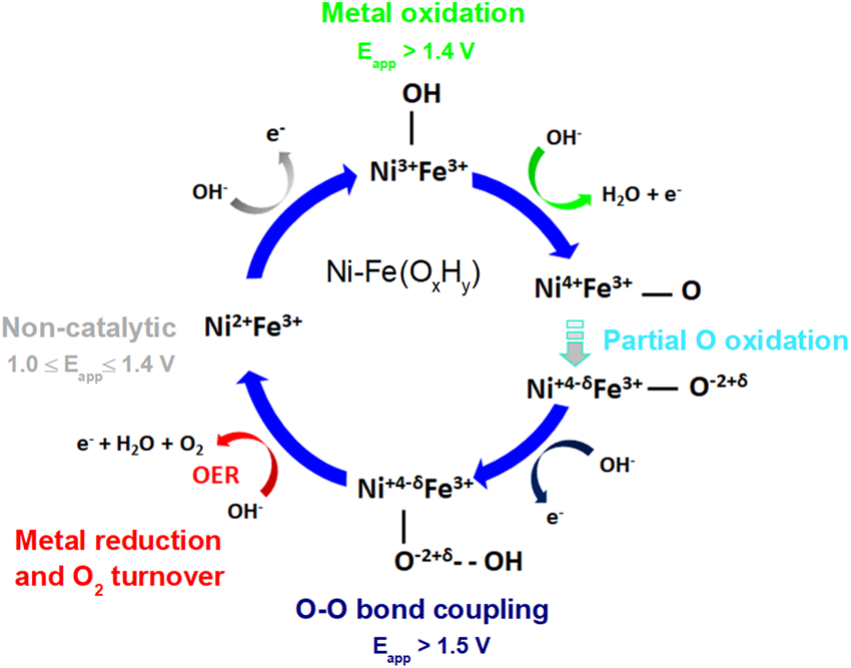
Mechanistic scheme summarizing the metal oxidation observed in our *in situ* XAS measurements of the Ni_65_Fe_35_(O_x_H_y_) catalyst. The resting state (non-catalytic potential) is observed between 1.0–1.4 V vs. RHE. Above this potential (>1.4 V) the oxidation of Ni^2+^ to Ni^3+/4+^ occurs. An oxidation state lower than +4 is usually observed both in Ni(OOH) and in mixed Ni-Fe(O_x_H_y_) catalysts. In the 1^st^ metal oxidation step (shown in green), a discharge of an OH^−^ group takes place, which adsorbs to a vacant site and increases the oxidation state by one unit. In this step a radical species may instead be formed (-OH∙) which would not result in a metal oxdation state increase (this is not shown in our cycle however may happen at both Fe and Ni sites). In the 2^nd^ metal oxidation step, Ni is further oxidized to Ni^4+^. However, if there is a consecuetive step where charge re-distribution occurs from O 2p → Ni 3d, the average metal oxidation state is lowered (Ni^+4-δ^), and as a consequence the oxygen oxidation state is increased (O^−2+δ^). This step according to our O *K*-edge data is a stable state and is strongly correlated to the metal oxidation step, and occurs prior to O-O bond formation.

What can be concluded from our soft XAS measurements –that adds new information for interpretation of the reaction intermediates occurring in the OER catalytic cycle - is that these O species appear and disappear in concomitance with the metal oxidation and reduction (Ni^2+^ ↔ Ni^3+/4^). Supported by work from other groups, this may be a more reactive O site that is electron deficient (partially oxidized) and of more radical character (denoted as O^−2+δ^), see proposed mechanistic scheme in Fig. 5.

We therefore consider this as an additional step in the mechanistic cycle that occurs at potentials prior to the onset of OER. In the first metal oxidation step in the OER cycle, coordination of a hydroxyl group (-OH) is shown. There is a possibility that a species of radical character is formed already in this step (-OH∙)^2,62,63^, which would not require an increase in formal oxidation state of the metal. Since the O species we observe are likely to be in its deprotonated form^60^, the formation of this species is more probable to follow after the 2^nd^ metal oxidation step which includes a deprotonation. This step occurs prior to the O-O bond coupling. We cannot distinguish whether the observed O species are located at Fe or Ni sites, however we have reasons to believe that they are closely associated with Ni sites owing to the large modulation seen at those sites according to our XAS data. Whether our observed O *K* species are distinct to the “active oxygen” species detected in Raman spectroscopic studies by Koper and coworkers^26,27^ or to the “negatively charged oxygen” ligands generated at Fe-centers, as proposed by us in previous work based on a non-Nernstian behavior of the redox couple is not entirely clear but not excluded^20^. Those changes as well all occurred in concomitance with the Ni^2+^ → Ni^3+/4+^ redox transition. This work hence demonstrates that all spectroscopic changes are well correlated with the metal oxidation state changes in the investigated Ni-Fe(O_x_H_y_) catalyst and occurs at pre-catalytic potentials. These all represents an equilibrium state well observed at room temperature conditions. It should be kept in mind that Fe ions may impact on the rate constants in the catalytic cycle of Ni-Fe catalysts. This may in turn introduce changes in the observed equilibrium states^7^. It was recently demonstrated by Goddard and coworkers in a theoretical study where Fe substitution in Ni(OOH) drastically changed the rate determining step, and thereby shifted the Ni^4+^ resting state in Ni(OOH) to a more reduced Ni^3+^ resting state in Ni-Fe(OOH). In recent work by Gray and coworkers it was shown that nonaqueous electrolyte may allow for spectroscopic detection of a broader range of metal oxidation states, where Fe atoms were observed to transit via a highly oxidized Fe^6+^ state in the OER catalytic cycle^22^.

As conclusion, the signature at ~529 eV in the O *K* spectra may indicate formation of partly electron deficient oxygen sites (O^−2+δ^) related to the formation of oxidized Ni^3+/4+^, and persist throughout OER catalytic as long as oxidized metal species prevail. This means that prior to the O-O bond formation step, the Ni-Fe(O_x_H_y_) catalyst has accumulated electron deficiencies. The true nature and role of these electropositive oxygen sites in the OER mechanism would need further in-depth investigations.

## Conclusions

In summary, we have demonstrated that *in situ* XAS in the soft and hard X-ray regimes can deliver in-depth information on the role of the oxygen in an electrodeposited Ni_65_Fe_35_(O_x_H_y_) catalyst during oxygen evolution conditions. By scrutinizing a feature seen at the O *K*-edge, we conclude that the appearance of a pre-peak at 529 eV occurs concomitant with changes at Ni *L*-edge peak 854.1 eV and Ni *K* and Fe *K*-edges, corresponding to the process formation of oxidized Ni^3+/4+^. By combining CV cycling and *in situ* sXAS, we further retrieve a more fine-tuned potential dependence of these entangled processes. We confirm that the O *K* pre-peak and the Ni oxidation occurs at pre-catalytic potentials prioir to O-O bond formation, and persists during OER catalysis as long as there is oxidized nickel. The pre-peak feature implies increased hybridization between O(2p) and Ni3(d) states, which has been proposed to induce charge transfer from O → Ni. This gives rise to an electron deficient oxygen site, which fits with previous assignment of the pre-peak at 529 eV to O^1−^. These sites are likely to arise from undercoordinated µ_1_-O and µ_2_-O sites, which are found at “edge” or “defect” sites of the oxyhydroxide layers. These sites may play an important role in the OER activity.

## Supplementary information

Supporting Information

## Data Availability

The datasets generated during and/or analysed during the current study are available from the corresponding author on reasonable request.
