# Supplementary material for: Uncovering The Role of Oxygen in Ni-Fe(OxHy) Electrocatalysts using In situ Soft X-ray Absorption Spectroscopy during the Oxygen Evolution Reaction
Source: Sci Rep. 2019 Feb 6;9:1532. doi: 10.1038/s41598-018-37307-x (PMC6365557; doi:10.1038/s41598-018-37307-x)
Supplement: Supplementary file 1 — Supporting Information [file 41598_2018_37307_MOESM1_ESM.docx]

**Supporting information**

Uncovering The Role of Oxygen in Ni-Fe(O_x_H_y_) Electrocatalysts using In Situ Soft X-ray Absorption Spectroscopy during the Oxygen Evolution Reaction

Dorian Drevon^1*^, Mikaela Görlin^2*^, Petko Chernev^2^, Lifei Xi^1^, Holger Dau^2^, Kathrin M. Lange^1,3^

^1^ Operando Characterization of Solar Fuel Materials, Institute for Solar Fuels, Helmholtz-Zentrum Berlin für Materialien und Energie GmbH, 12489 Berlin, Germany

^2^ Free University of Berlin, Department of Physics, Arnimallee 14, 14195 Berlin, Germany

3 Universität Bielefeld, Physikalische Chemie, Universitätsstr. 25, 33615 Bielefeld, Germany

* these authors contributed equally to this work

Contents

[S1) Scanning electron microscopy (SEM) characterization 2](#_Toc511290761)

[S2) OER activity of the Ni-Fe catalyst 3](#_Toc511290762)

[S3) X-ray absorption spectroscopy at the Ni and Fe *K*-edges 4](#_Toc511290763)

[S4) Depedence of the absorption coefficient on applied potential at the O *K*-edge. 5](#_Toc511290764)

[S5) Multi peak fitting with Gaussian functions 6](#_Toc511290765)

[S6) Literature peak assignment 7](#_Toc511290766)

[S7) Reference spectra in the sXAS setup 9](#_Toc511290767)

[S8) In situ XAS and activity during CV cycling 10](#_Toc511290768)

[References 11](#_Toc511290769)

# Scanning electron microscopy (SEM) characterization


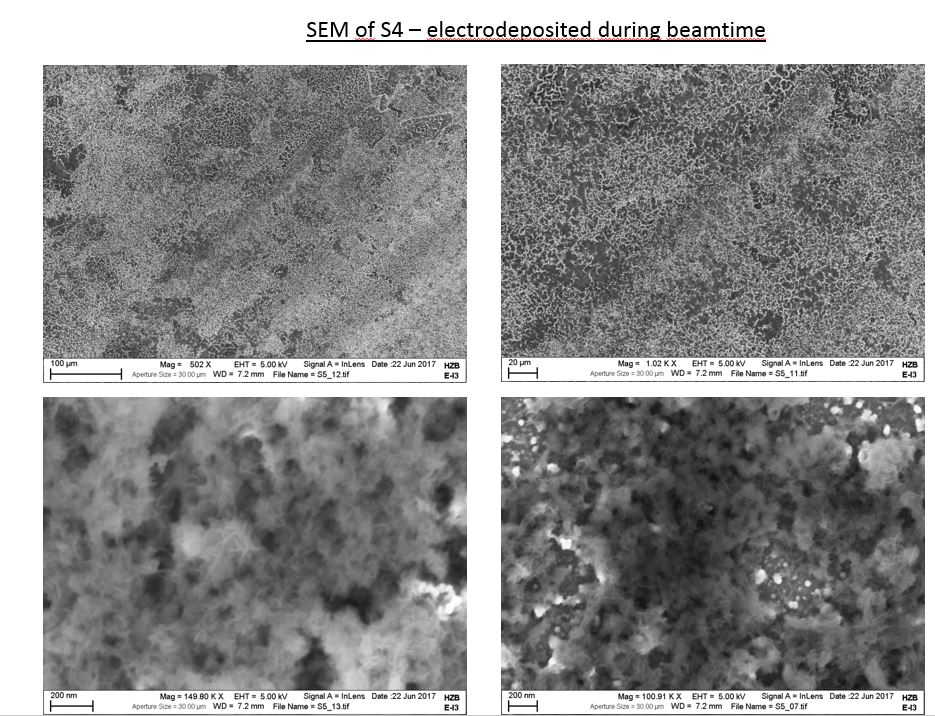


**Figure S1.** SEM images of electrodeposited Ni_65_Fe_35_(O_x_H_y_) catalysts on Si_3_N_4_/Ti/Au substrate after OER characterization at the O *K* and *L* edges at different electrode potentials in 0.1 M KOH.

# OER activity of the Ni-Fe catalyst


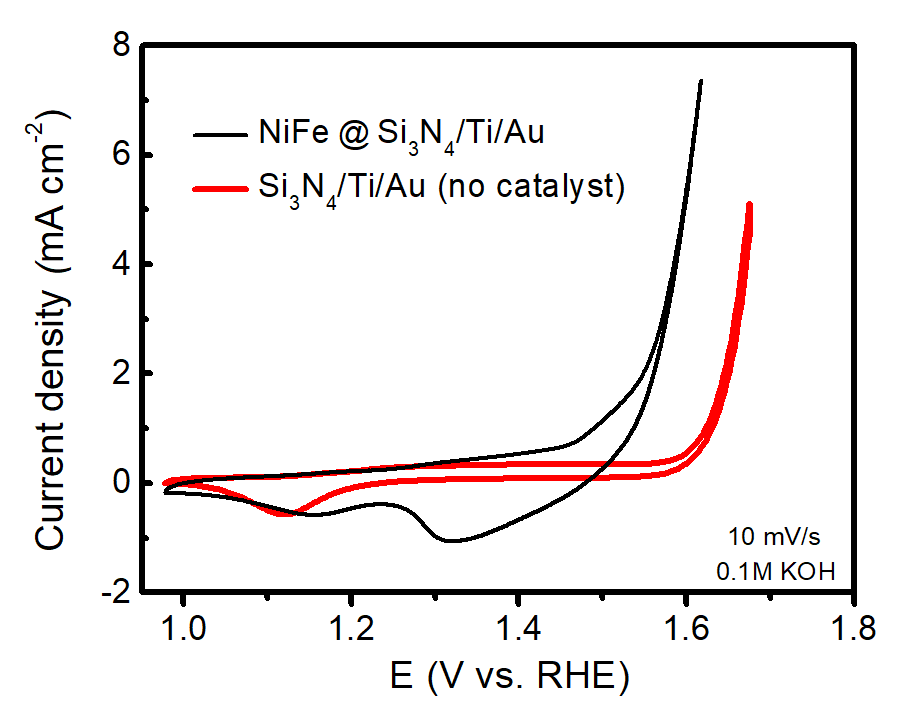


**Figure S2.** Comparison of an empty Si_3_N_4_/Ti/Au electrode substrate and a Si_3_N_4_/Ti/Au electrode with electrodeposited Ni_65_Fe_35_(O_x_H_y_) catalyst. The CVs were recorded in 0.1 M KOH at a scan-rate of 10 mV/s.


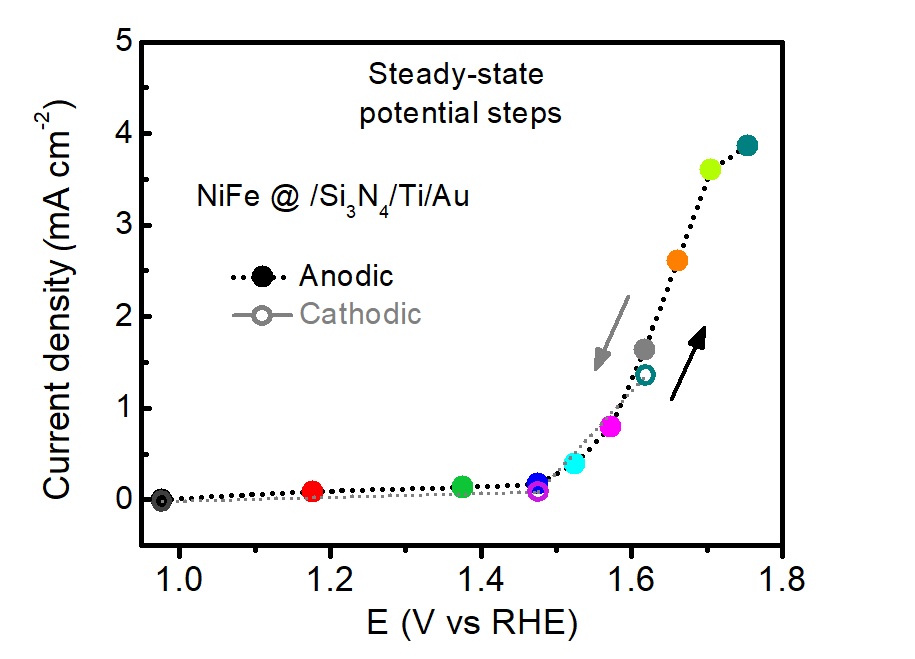


**Figure S3.** Steady-state measurements of the electrodeposited Ni_65_Fe_35_(O_x_H_y_) catalyst in 0.1 M KOH showing the current density at different electrode potentials during acquisition of the X-ray absorption O *K*- and *L*-edges. The working electrode was a Si_3_N_4_/Ti/Au electrode substrate.

# X-ray absorption spectroscopy at the Ni and Fe *K*-edges


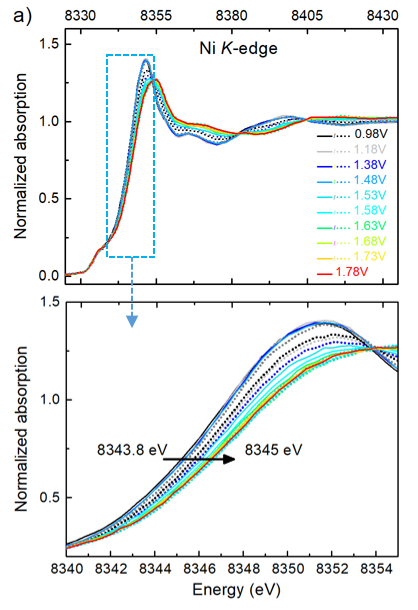

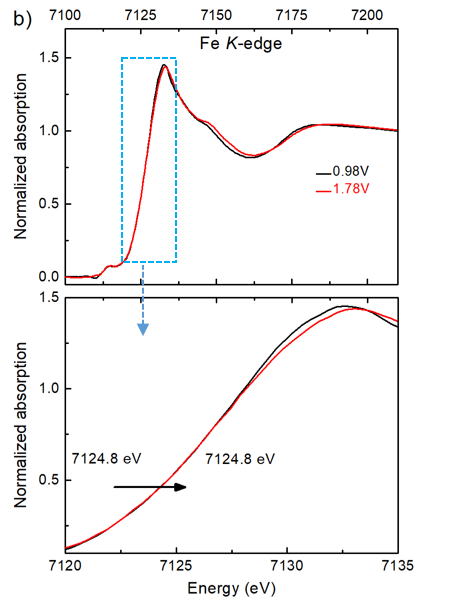


**Figure S4.** In situ X-ray absorption spectra at the *K*-edges of the Ni_65_Fe_35_(O_x_H_y_) catalyst on a GC-Au electrode. The spectra were recorded during steady-state conditions. **(a)** Ni *K*-edge at various electrode potentials; forward steps and increasing potential (*solid lines*), and the reverse potential steps (*dashed lines*)*.* **(b)** Fe *K*-edge at 0.98 V and 1.78 V vs. RHE. The measurements were carried out in 0.1 M KOH.

# *Linear combination*


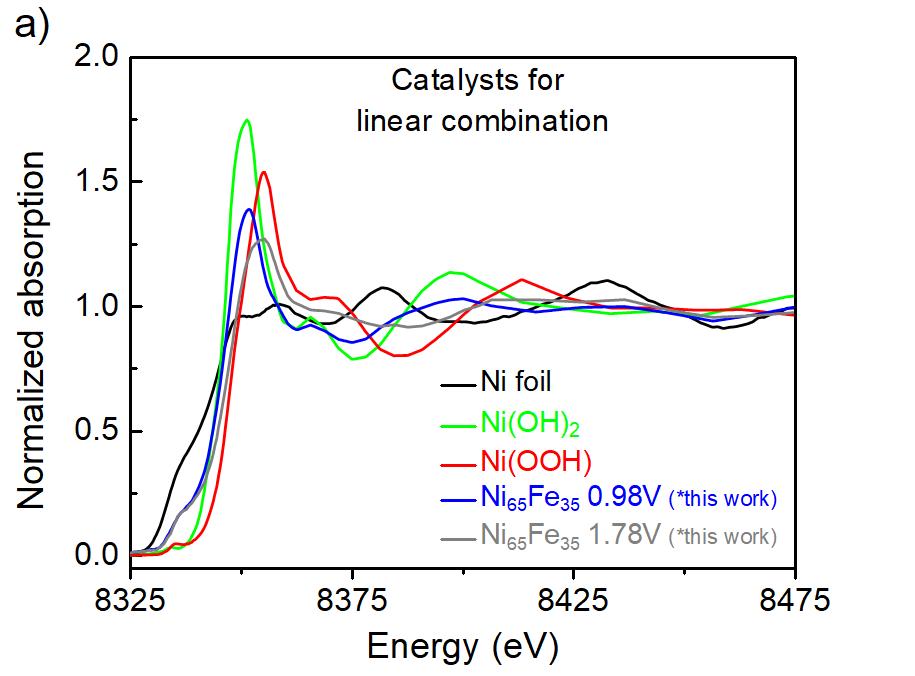

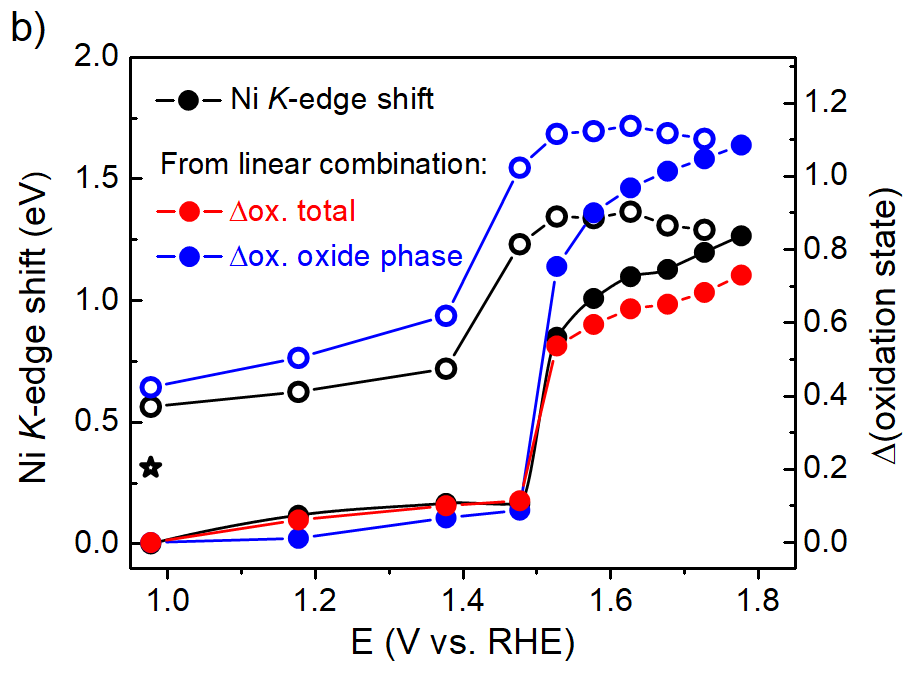


**Figure S5.** In situ XAS measurements at the Ni *K*-edge **(a)** References used for the linear combination fitting, metallic Ni-foil, Ni(OH)_2_, Ni(OOH), and the Ni_65_Fe_35_ catalyst at 0.98 V and 1.78 V vs. RHE (*this work). **(b)** The Ni *K*- edge shift with respect to the edge position at 0.98 V (*black dots*, *left axis*) and the change in oxidation state (Δox., *right axis*) of the total phase (*red*) and of the oxide phase (*blue*) after subtraction of the metallic contribution determined by linear combination*.* The stars shows the edge shift after an additional waiting time of ~10 min after returning to 0.98 V from OER catalytic potentials. The measurements were carried out in 0.1 M KOH.

# Depedence of the O *K*-edge absorption coefficients on potential

*
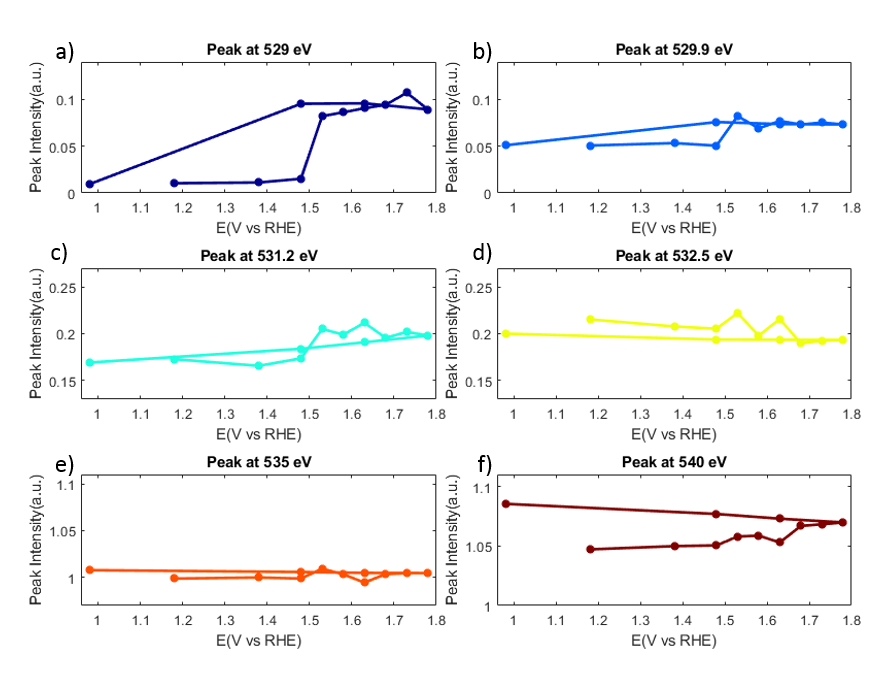
*

**Figure S6.** Dependence on the applied electrode potential of the area of the Gaussian curves used for multi-peak fitting at the O K pre-edge region of an electrodeposited Ni_65_Fe_35_ catalyst **(a)** 529 eV peak **(b)** 529.9 eV **(c)** 531.2 eV **(d)** 532.5 eV **e)** 535 eV **f)** 540 eV. The measurements were carried out in 0.1 M KOH.

# Multi peak fitting with Gaussian functions

The water pre-peak at 535 eV was first estimated with a Gaussian function. It was then subtracted from the spectrum to isolate the metal region before 535 eV. A Gaussian peak is defined by three parameters: the height, the position and the full width at half maximum (FWMH). The *position* corresponds to the average of the pdf. The relation between the *FWMH* and the standard deviation $\sigma$ is: $FWMH=\sigma*2\sqrt{2*ln2}$. The area is the integral of the peak.

**Table S1.** Gaussian fit parameters at a potential of 1.18 V vs. RHE

| **Peak** | **Position (eV)** | **height** | **FWMH** | **area** |
| --- | --- | --- | --- | --- |
| Ni(3d)t_2g_ | 529.0 | 0.00 | - | 0.00 |
| Fe(3d)t_2g_ | 529.9 | 0.05 | 0.83 | 0.04 |
| O($\pi$*) of O_2_ | 531.2 | 0.16 | 1.05 | 0.18 |
| Fe(3d)e_g_ | 532.5 | 0.15 | 1.25 | 0.20 |

**Table S2.** Gaussian fit parameters at a potential of 1.78 V vs. RHE

| **Peak** | **Position (eV)** | **height** | **FWMH** | **area** |
| --- | --- | --- | --- | --- |
| Ni(3d)t_2g_ | 529.0 | 0.09 | 1.03 | 0.10 |
| Fe(3d)t_2g_ | 530.0 | 0.06 | 0.85 | 0.05 |
| O($\pi$*) of O_2_ | 531.2 | 0.18 | 1.08 | 0.21 |
| Fe(3d)e_g_ | 532.7 | 0.15 | 1.67 | 0.27 |

# Literature peak assignment

**Table S3.** Literature of the pre-feature at the O K-edge for selected Ni and Fe compounds. If no values are given the information was not reported in the reference.

| **Sample** | **Peak Position(eV)** | **Energy calibration(eV)** | **delta energy (eV)** | **feature used for calibration** | **Peak Position after calibration(eV)** | **Orbital hybridization O2p with** | **Reference** |
| --- | --- | --- | --- | --- | --- | --- | --- |
| NiO | 528.5 | No energy calibration | -0.5 | NiO at 540 | 528 | t_2g_ | Cho 2013^1^ |
| NiO | 533 | No energy calibration | -0.5 | NiO at 540 | 532.5 | Ni^2+^ | Cho 2013^1^ |
| NiO | 530 | No energy calibration | -0.5 | NiO at 540 | 529.5 | multiplet | Cho 2013^1^ |
| LiNi1-xAlxO2 | 525.5 | LiCoO2 pre –edge at 527.5 eV | - | - | - | e_g_ | Kim 2004^2^ |
| LiNiCoMnO | 526.2 | L-edge of Metal foil | - | - | - | Ni(III)O | Kim 2005^3^ |
| LiNiCoMnO | 527.3 | L-edge of Metal foil | - | - | - | Ni(II)O | Kim 2005^3^ |
| NiBi | 528.7 | 530.8 O1s-pi* of O2 gas | 0.4 | 530.8 O1s-π* of O_2_ gas | 529.1 | e_g_ | Yoshida 2015^4^ |
| NiFeO_x_/C | 527.7 | No energy calibration | - | - | - | metal 3d | Wang 2015^5^ |
| NiFeO_x_/C | 532.7 | No energy calibration | - | - | - | Ni3d states | Wang 2015^5^ |
| Fe_2_O_3_ | 530 | Cr powder at L3 574.1 eV | -0.4 | Fe_2_O_3_ t_2_g at O K-edge | 529.6 | t_2g_ | Hayes 2011^6^ |
| Fe_2_O_3_ | 531.5 | Cr powder at L3 574.1 eV | -0.4 | Fe_2_O_3_ t_2_g at O *K*-edge | 531.1 | e_g_* | Hayes 2011^6^ |
| LaFeO_3_ | 530 | Cr powder at L3 574.1 eV | -0.4 | Fe_2_O_3_ t_2_g at O *K*-edge | 529.6 | t_2g_ | Hayes 2011^6^ |
| LaFeO_3_ | 531.5 | Cr powder at L3 574.1 eV | -0.4 | Fe_2_O_3_ t_2_g at O *K*-edge | 531.1 | e_g_ * | Hayes 2011^6^ |
| LaFeO_3_ | 530 | done but unclear | 0.4 | hayes LaFeO_3_ | 530.4 | 3d(pi sym t2g) | Lafuerza 2011^7^ |
| LaFeO_3_ | 531.5 | done but not say how | 0.4 | hayes LaFeO_3_ | 531.9 | e_g_* | Lafuerza 2011^7^ |
| LaFeO_3_ | 530 | No energy calibration | 0.4 | hayes LaFeO_3_ | 530.4 | t_2g_ | Suntivitch 2014^8^ |
| LaFeO_3_ | 531.5 | No energy calibration | 0.4 | hayes LaFeO3 | 531.9 | e_g_ | Suntivitch 2014^8^ |
| NiFeO_3_ | 529.2 | No energy calibration | 0.4 | hayes LaFeO3 | 529.6 | e_g_ | Suntivitch 2014^8^ |
| FePO_4_ | 531.8 | No energy calibration | - | - | - | Fe 3d | Augustsson 2005^9^ |
| FeOOH | 529.8 | No energy calibration | - | - | - | Fe 3d | Gilbert 2007^10^ |
| FeOOH | 531 | No energy calibration | - | - | - | Fe 3d | Gilbert 2007^10^ |
| Fe_2_O_3_ | 525.75 | verified with NiO | 2 | H_2_O | 527.75 | t_1_u | Guo 2012^11^ |
| Fe_2_O_3_ | 527.08 | verified with NiO | 2 | H_2_O | 529.08 | A_1_g | Guo 2012^11^ |
| Fe_2_O_3_ | 529 | verified with NiO | 2 | H_2_O | 531 | t_2g_ | Guo 2012^11^ |
| Fe_2_O_3_ | 530.1 | verified with NiO | 2 | H_2_O | 532.1 | e_g_ | Guo 2012^11^ |
| NiFe | 530.1 | - | 1.75 | H_2_O | 531.85 | M-O | Ali-Llöytty 2016^12^ |
| NiFe | 531.6 | - | 1.75 | H_2_O | 533.35 | M-OH | Ali-Llöytty 2016^12^ |
| La_2_NiO_4_ | 528.5 | No energy calibration | - | - | - | eg | Nakamura 2016^13^ |
| La_2_Ni_1_-xFexO_4_ | 530 | No energy calibration | - | - | - | t_2g_ | Nakamura 2016^13^ |

# Reference spectra in the sXAS setup


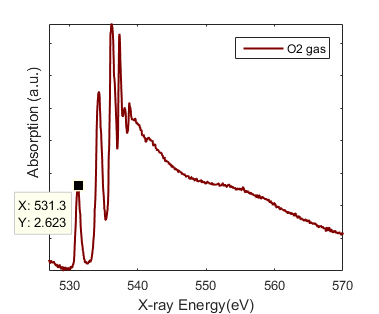


**Figure S7.** Reference spectrum of O_2_ gas at the O *K*-edge, recorded in the in situ cell sXAS cell.


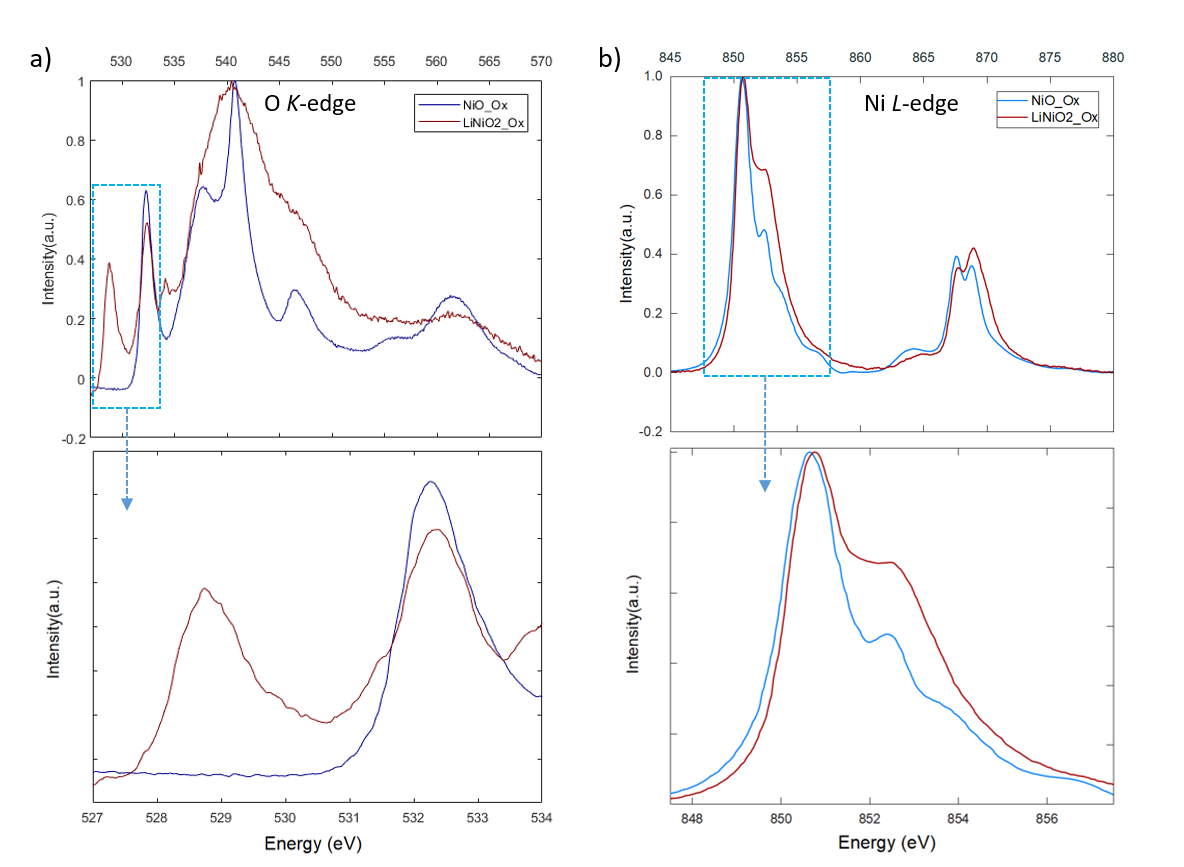


**Figure S8.** **(a)** O *K*-edge spectra of powder reference compounds NiO and LiNiO_2_ (*top*). The bottom figure shows a zoom in of the marked pre-edge region. **(b)** Ni *L*-edge spectra of NiO and LiNiO_2_ (*top*). The bottom figure shows the marked region.

# In situ XAS and activity during CV cycling


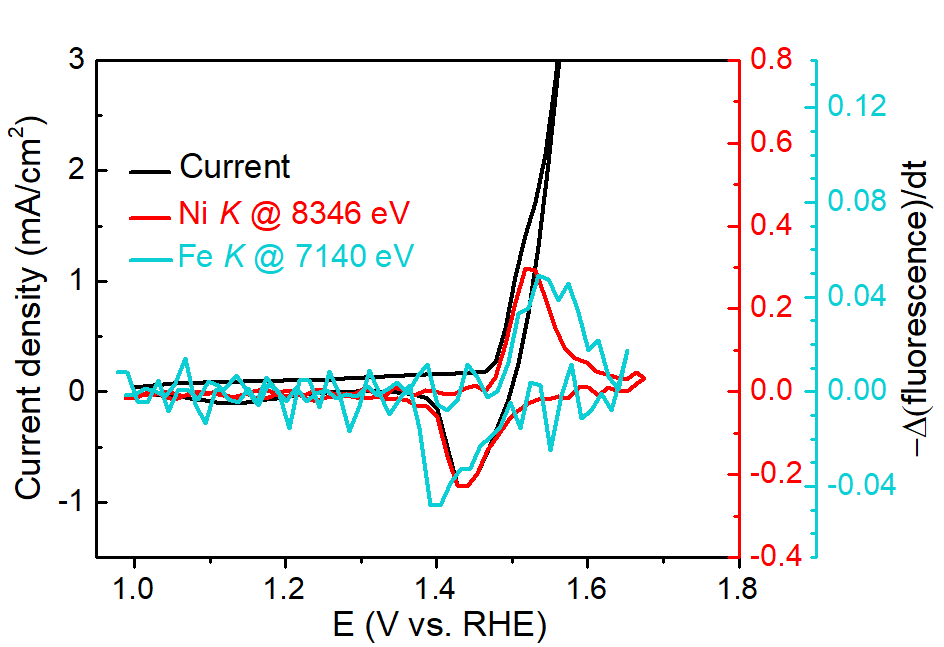


**Figure S9.** In situ XAS at the Ni and Fe *K*-edges of the electrodeposited Ni_65_Fe_35_(O_x_H_y_) catalyst while cycling a CV between 0.98-1.68 V vs. RHE. The fluorescence signals at the Ni and Fe *K*-edges at a fixed beam energy (8346 eV for Ni *K* and 7140 eV for Fe *K*) monitored during the CV sweep. The negative derivative of the fluorescence signal in was then determined. The All measurements were carried out in 0.1 M KOH. The working electrode was an Au-coated glassy carbon electrode (GC-Au) with an area of 1.96 cm^2^.

# References

1. Cho, D.-Y. *et al.* Spectroscopic investigation of the hole states in Ni-deficient NiO films. *J. Mater. Chem. C* **1,** 4334 (2013).

2. Kim, M. G. *et al.* Ni and oxygen K-edge XAS investigation into the chemical bonding for lithiation of LiyNi1−xAlxO2 cathode material. *Electrochim. Acta* **50,** 501–504 (2004).

3. Kim, M. G., Shin, H. J., Kim, J.-H., Park, S.-H. & Sun, Y.-K. XAS Investigation of Inhomogeneous Metal-Oxygen Bond Covalency in Bulk and Surface for Charge Compensation in Li-Ion Battery Cathode Li[Ni[sub 1∕3]Co[sub 1∕3]Mn[sub 1∕3]]O[sub 2] Material. *J. Electrochem. Soc.* **152,** A1320 (2005).

4. Yoshida, M. *et al.* Direct Observation of Active Nickel Oxide Cluster in Nickel–Borate Electrocatalyst for Water Oxidation by In Situ O K-Edge X-ray Absorption Spectroscopy. *J. Phys. Chem. C* **119,** 19279–19286 (2015).

5. Wang, D. *et al.* In Situ X-ray Absorption Near-Edge Structure Study of Advanced NiFe(OH) x Electrocatalyst on Carbon Paper for Water Oxidation. *J. Phys. Chem. C* **119,** 19573–19583 (2015).

6. Hayes, J. R. & Grosvenor, A. P. An x-ray absorption spectroscopic study of the electronic structure and bonding of rare-earth orthoferrites. *J. Phys. Condens. Matter* **23,** 465502 (2011).

7. Lafuerza, S. *et al.* Origin of the pre-peak features in the oxygen K-edge x-ray absorption spectra of LaFeO 3 and LaMnO 3 studied by Ga substitution of the transition metal ion. *J. Phys. Condens. Matter* **23,** 325601 (2011).

8. Suntivich, J. *et al.* Estimating Hybridization of Transition Metal and Oxygen States in Perovskites from OK-edge X-ray Absorption Spectroscopy. *J. Phys. Chem. C* **118,** 1856–1863 (2014).

9. Augustsson, A. *et al.* Electronic structure of phospho-olivines LixFePO4 (x=0,1) from soft-x-ray-absorption and -emission spectroscopies. *J. Chem. Phys.* **123,** 184717 (2005).

10. Gilbert, B. *et al.* Oxygen K-Edge Emission and Absorption Spectroscopy of Iron Oxyhydroxide Nanoparticles. in *AIP Conference Proceedings* **882,** 721–725 (AIP, 2007).

11. Guo, J. *et al.* Direct Observation of Two Electron Holes in a Hematite Photoanode during Photoelectrochemical Water Splitting. *J. Phys. Chem. C* **116,** 16870–16875 (2012).

12. Ali-Löytty, H. *et al.* Ambient-Pressure XPS Study of a Ni–Fe Electrocatalyst for the Oxygen Evolution Reaction. *J. Phys. Chem. C* **120,** 2247–2253 (2016).

13. Nakamura, T., Oike, R., Ling, Y., Tamenori, Y. & Amezawa, K. The determining factor for interstitial oxygen formation in Ruddlesden–Popper type La 2 NiO 4 -based oxides. *Phys. Chem. Chem. Phys.* **18,** 1564–1569 (2016).
